# Individual-level determinants of late-stage cervical cancer diagnosis and their implications for prevention and control

**DOI:** 10.3332/ecancer.2025.2008

**Published:** 2025-10-07

**Authors:** Adeniyi K Akiseku, Taiwo O Adenuga, Olusoji E Jagun, Mutiu A Popoola, Adetola O Olatunji

**Affiliations:** 1 Department of Obstetrics and Gynaecology, Olabisi Onabanjo University, Ago Iwoye, Ogun State 120107, Nigeria; 2 Department of Obstetrics and Gynaecology, Olabisi Onabanjo University Teaching Hospital, Ago Iwoye, Ogun State 121101, Nigeria; a https://orcid.org/0000-0003-4530-6479

**Keywords:** cervical cancer, late-stage diagnosis, education, menopausal status, low-income countries, Nigeria

## Abstract

**Background:**

Cervical cancer remains a significant public health issue, particularly in low-income countries. It is the fourth most common cancer among women globally, with an estimated 570,000 new cases and 311,000 deaths in 2018.

**Objective:**

This study aimed to examine the stages of cervical cancer at diagnosis and identify factors contributing to late-stage presentation among women in a tertiary care hospital in Nigeria.

**Methods:**

A retrospective study analysed data from women diagnosed with cervical cancer between 2017 and 2021. Demographic, reproductive and clinical data were extracted from medical records.

**Results:**

Of the 102 women who presented during the study period, only 57 (55.9%) had complete staging, clinical and demographic data; these complete cases were included to ensure data integrity. From this population, 73.7% were aged 50 years or older and 56.1% presented with late-stage disease. Additionally, anaemia (packed cell volume <30%) was present in 75.4% of women. Postcoital bleeding was reported in 35.1% of cases. Women with no formal education had higher odds of late-stage diagnosis odds ratios (OR: 4.40, 95% CI: 1.08–17.82). Postmenopausal women also had higher odds of late-stage diagnosis (OR: 4.46, 95% CI: 1.27–15.70).

**Conclusion:**

A late-stage cervical cancer diagnosis is prevalent among women in Nigeria, particularly among those with lower educational levels and postmenopausal women. Targeted awareness programmes, expanded screening (including integration into well-woman/postmenopausal care) and improved healthcare infrastructure, including consistent documentation of screening history and human papillomavirus vaccination, are essential for reducing the burden of cervical cancer in this context.

## Introduction

Cervical cancer remains one of the most pressing public health challenges globally, particularly in low-income countries (LICs) [1,2]. It is the fourth most common cancer among women worldwide, with an estimated 570,000 new cases and 311,000 deaths in 2018 [3–5]. Despite advancements in cancer prevention, diagnosis and treatment, cervical cancer continues to disproportionately affect LICs due to limited healthcare resources and infrastructure [5–7]. Research in sub-Saharan Africa has revealed alarming mortality rates, with cumulative deaths ranging from 65% to 68% over a 2–5-year period [8–10]. In Nigeria specifically, the 5-year cervical cancer mortality prevalence was estimated at 22.11 per 100,000 women [11], accounting for 14.8% of all cancer-related deaths among Nigerian women in 2018 [12].

The primary etiological factor for cervical cancer is persistent infection with high-risk human papillomavirus (HPV) types, particularly HPV-16 and HPV-18, which are responsible for approximately 70% of all cases [1, 5, 13]. In high-income countries, organised screening programs using Pap smears and HPV testing have led to substantial reductions in cervical cancer incidence and mortality [5, 14, 15]. However, in LICs, the implementation of cervical cancer prevention programs is hindered by inadequate healthcare infrastructure, insufficient healthcare personnel, limited public awareness, inadequate health funding and poor healthcare financing mechanisms, particularly the lack of comprehensive health insurance coverage [10, 14–16]. Consequently, cervical cancer is often diagnosed at advanced stages in these regions, leading to poorer prognoses and higher mortality rates [10, 14]. To combat this, the World Health Organisation (WHO) is developing a global plan of action to engage stakeholders and mobilise resources to make cervical cancer a rare disease globally [15, 17, 18]. Nigeria has also taken steps to introduce the HPV vaccine into its routine immunisation system, aiming to reach 7.7 million girls aged 9–14 years [13, 19].

The International Federation of Gynaecology and Obstetrics (FIGO) staging system is the most widely used framework for classifying the extent of cervical cancer. It ranges from stage I, where the cancer is confined to the cervix, to stage IV, where it has spread to distant organs [5, 14, 20]. The treatment approach for cervical cancer depends on the stage at diagnosis, with early-stage cases typically managed with surgery or radiotherapy, while advanced stages often require a combination of radiotherapy and chemotherapy [5, 14, 20].

Studies have shown that in many LICs, a substantial proportion of women present with advanced-stage disease [1, 2, 21]. Studies in Uganda, Ghana and Nigeria showed that a significant proportion of cervical cancer patients presented at late stages, with 61% in Uganda, 67.2% in Ghana and 72.8% in Nigeria diagnosed at advanced stages [14, 21, 22]. This late presentation is being attributed to socioeconomic barriers, cultural beliefs and inadequate access to healthcare services [14].

This study examines the stages of cervical cancer in women when they first arrive at a tertiary care hospital. By analysing the stages at diagnosis and identifying factors that contribute to late-stage presentation, the study aims to explain strategies for early detection and reduce the burden of cervical cancer, particularly in vulnerable populations.

## Methods

This retrospective study analysed data from women diagnosed with cervical cancer at a tertiary care hospital in Nigeria between January 2017 and December 2021. Inclusion criteria consisted of women aged 18 years or older with a confirmed histopathologic diagnosis of cervical cancer and complete staging information. Women with incomplete reproductive history or clinical staging data, as well as those with a history of prior cancer treatment, were excluded.

Data extraction involved reviewing medical records to collect demographic information (age, marital status), reproductive history variables (parity, age at menarche and age at coitarche) and clinical data (FIGO staging, presence of postcoital bleeding, menopause status and anaemia based on packed cell volume (PCV) levels). The primary outcome was the stage of cervical cancer at diagnosis, categorised as early-stage (stages I–IIA) or late-stage (stages IIB–IV) according to the FIGO 2018 classification [20].

### Data analysis

Descriptive analysis: The distributions of reproductive variables, including parity, age at menarche and age at coitarche, were summarised using means, medians and standard deviations. FIGO cancer stages were categorised as early-stage (Stages One and Two) or late-stage (Stages Three and Four). Inferential statistical analyses were conducted to assess the factors influencing the likelihood of late-stage cervical cancer diagnosis. Specifically, odds ratios (ORs) and adjusted odds ratios (AORs) were calculated to determine the strength and direction of associations between key variables (such as parity, age at menarche and age at coitarche) and cancer stage at diagnosis.

### Statistical software

All statistical analyses were conducted using SPSS version 25.0 (IBM Corp., Armonk, NY) and statistical significance was set at *p*-values less than 0.05.

## Results

Of the 102 women who presented to the clinic during the study period, only 57 (55.9%) had complete demographic and clinical data records from the Obstetrics and Gynaecology Department of the Institution. We restricted analyses to these 57 cases to maintain data integrity and reliable statistical analysis. The participants' ages ranged from 27 to 85 years, with a mean age of 57.23 years and a median age of 55. Parity varied from 0 to 11 children, with a median of 5 children. The age at menarche ranged from 9 to 21 years, with a median of 15 years and a mean of 14.75 years, while the age of first sexual intercourse (coitarche) varied between 15 and 30 years, with a median of 19 years and a mean of 19.67 years.

Table 1 revealed that 73.7% of participants were 50 years or older, and a significant proportion (40.4%) had between 5 and 7 children. The most common age at first sexual intercourse was between 16 and 19 years, accounting for 43.9% of cases. Additionally, 59.6% of participants had their first menstruation between the ages of 11 and 15 years. The majority of women were traders (70.1%), while artisans, civil servants, clergy, unemployed individuals and teachers each represented less than 10% of the cohort. Marital status analysis showed that nearly 60% of participants were married at the time of diagnosis. Regarding education levels, 24.6% had no formal education, 21.0% completed primary education, 38.6% attained secondary education and 15.8% had tertiary education.

**Figure 1.**
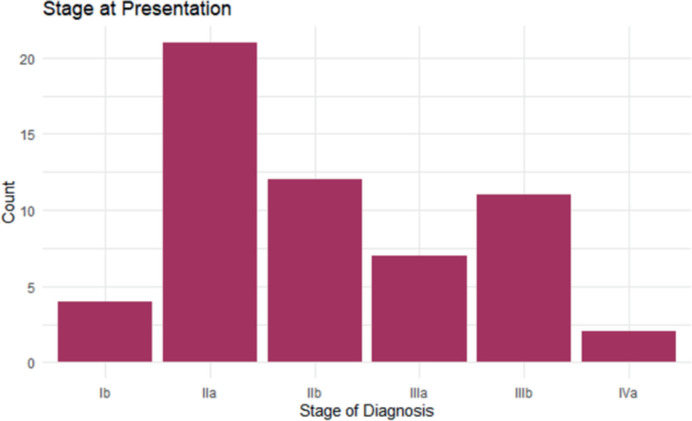
Bar chart showing stages of disease at presentation.

Clinical distribution can be seen in Table 2, which indicates that 56.2% of cases were diagnosed at an advanced stage (stages IIB and IV), Figure 1. Additionally, anaemia (PCV <30%) was present in 75.4% of women. Postcoital bleeding was reported in 35.1% of cases. Most participants (73.7%) were postmenopausal at the time of diagnosis.

Analysis of associations revealed that women with no formal education had significantly higher odds of late-stage cervical cancer diagnosis compared to those with tertiary education (OR: 4.40, 95% CI: 1.08–17.82; AOR: 3.20, 95% CI: 1.02–10.05). Postmenopausal women were also significantly more likely to present with late-stage cervical cancer compared to premenopausal women (OR: 4.46, 95% CI: 1.27–15.70; AOR: 3.80, 95% CI: 1.25–11.55).

Several trends did not reach statistical significance but suggested potential associations. Women with coitarche at ages 16–19 had higher odds of late-stage diagnosis compared to those aged ≥30, though this was not statistically significant (OR: 2.41, 95% CI: 0.81–7.17; AOR: 1.90, 95% CI: 0.70–5.15). Similarly, women with a PCV of 30 or higher had increased odds of late-stage cancer (OR: 2.75, 95% CI: 0.76–10.02; AOR: 2.50, 95% CI: 0.70–9.50), though these findings were not significant (Tables 3 and 4).

## Discussion

The present study provides a detailed analysis of demographic, clinical and associated risk factors for cervical cancer among women diagnosed at our institution. The findings are consistent with global trends in cervical cancer epidemiology, highlighting key associations between late-stage diagnosis and factors such as education level and menopausal status.

The mean age of the participants was 57.2 years, with 73.7% of cases occurring in women aged 50 years or older. This age distribution aligns with studies indicating that cervical cancer predominantly occurs in this age group in LIC [2, 21, 23–25]. However, a concerning trend has emerged globally, with an increasing incidence of cervical cancer among young women aged 15–49 years,

particularly in areas with high Socio-Demographic Index scores. This upward trend may be attributed to various factors, including increased exposure to HPV, lower participation rates in cervical cancer screening programs, earlier sexual debut and a history of multiple sexual partners [17, 26].

A substantial proportion of participants (40.4%) had between 5 and 7 children. High parity has been widely recognised as a risk factor for cervical cancer, likely due to prolonged exposure to HPV, cervical trauma and hormonal changes during pregnancy [4, 23, 27]. In contrast, the age at menarche and coitarche did not demonstrate statistically significant associations with late-stage diagnosis, despite previous studies suggesting an increased risk with earlier sexual debut [28, 29].

Late-stage cervical cancer (FIGO stages IIB–IV) was observed in 56.1% of cases. This is comparable to findings from other low-resource settings, where delayed diagnosis is frequently reported due to limited access to screening programs [2, 14, 21, 25]. A study conducted in Ethiopia similarly reported that over 60% of women presented with advanced-stage disease due to inadequate screening and late health-seeking behaviour [30]. Inadequacies in the healthcare system may also play a significant role in the late-stage diagnosis of cervical cancer [2, 26, 30]. Additionally, prolonged investigation times and facility-based delays can further hinder early diagnosis and timely initiation of appropriate care [21, 22].

Anaemia was present in 75.4% of cases, highlighting the significant nutritional and systemic health challenges faced by cervical cancer patients in resource-limited settings. Reports indicate that 40%–64% of cervical cancer patients presenting for treatment are anaemic, with some studies showing rates exceeding 80% at diagnosis [2, 31]. Notably, anaemia is approximately twice as prevalent in patients with advanced-stage cervical cancer compared to those diagnosed at an earlier stage [31]. This, however, differs from what was seen in this study, which showed marginal differences. This high prevalence of anaemia in cervical cancer patients can be attributed to multiple factors, including tumour-related bleeding, bone marrow invasion, malnutrition, disrupted iron metabolism, renal impairment and compromised bone marrow function [2, 31, 32].

Women with no formal education were significantly more likely to be diagnosed at an advanced stage (OR = 4.4, *p* = 0.045). After adjusting for confounders, the odds remained high (AOR = 3.20, 95% CI: 1.02–10.05). This finding supports previous studies linking lower educational attainment to delayed diagnosis, as education influences health literacy, awareness of cervical cancer symptoms and healthcare-seeking behaviour [21, 33]. Notably, in a systematic review, women with low or no formal education are more likely to present with late-stage cervical cancer [34]. The findings highlight the urgent need for targeted awareness programs to improve cervical cancer screening uptake among women with lower educational backgrounds. Furthermore, the lack of education is often linked to early marriage and high parity, which are independent risk factors for cervical cancer [35]. Therefore, improving women's literacy levels can be a crucial strategy in controlling cervical cancer, emphasising the importance of education in cancer prevention.

From this study, postmenopausal women had 4.5 times higher odds of late-stage diagnosis (*p* = 0.014), which remained significant after adjustment (AOR = 3.80, 95% CI: 1.25–11.55). The significantly higher odds of late-stage diagnosis among postmenopausal women may be attributed to reduced engagement with gynaecological care after menopause. Younger women, due to their reproductive health needs, tend to have more frequent interactions with healthcare providers, facilitating earlier detection [21]. A Vietnamese study further asserted that the postmenopausal state increases the susceptibility to late-stage presentation as a result of hormonal and immunological changes [27]. The establishment of well-woman clinics can significantly improve early detection rates, ultimately leading to a lower prevalence of late-stage cervical cancer.

Eliminating cervical cancer is possible with high-coverage screening and vaccination, yet progress will likely be slowest in low-income regions, particularly in sub-Saharan Africa, due to existing healthcare gaps [36]. To combat this, LICs must strengthen health systems to support widespread screening and vaccination efforts. Achieving the WHO's global strategy targets requires significant investment in healthcare infrastructure, capacity building and public engagement [36–38].

## Implications and recommendations

Targeted awareness campaigns: Develop literacy-focused educational interventions (for example, radio programmes and community workshops) aimed at women with low or no formal education to increase screening uptake.

Integration of screening into postmenopausal care: Incorporate Pap smears and HPV testing into routine care for postmenopausal women (well-woman clinics) to minimise delays in diagnosis.

Enhance health infrastructure: Ensure a reliable supply of screening equipment, consistent documentation of screening history and HPV vaccination and develop capacity for healthcare workers in early detection protocols.

Future research directions: Conduct multicentre, mixed-methods studies to explore cultural, socioeconomic and health system barriers in greater depth.

In conclusion, this study's findings highlight the persistent challenges in cervical cancer diagnosis and treatment in low-income settings. The results show that late-stage diagnosis is prevalent. Significant associations were found between late-stage diagnosis and factors such as lower educational attainment and postmenopausal status. The study's findings emphasise the need for targeted interventions, including improved access to screening and education, particularly for high-risk groups. By addressing these disparities and strengthening healthcare systems, progress can be made towards eliminating cervical cancer in low-income settings.

## List of abbreviations

AOR, Adjusted odds ratios; FIGO, The International Federation of Gynaecology and Obstetrics; HPV, Human papillomavirus; LICs, Low-income countries; OR, Odds ratios; WHO, World Health Organisation.

## Conflicts of interest

The authors declare that they have no competing interests.

## Funding

This study was not funded.

## Consent for publication

Not applicable.

## Figures and Tables

**Table 1.** Demographic distribution.

Category	Interval	Count	Percentage
Age	20–29	1	1.8%
	30–39	4	7.0%
	40–49	10	17.5%
	50–59	20	35.1%
	≥60	22	38.6%
Parity	0–1	3	5.3%
	2–4	20	35.1%
	5–7	23	40.4%
	≥8	11	19.3%
PCV distribution	≤19	17	29.8%
	20–29	26	45.6%
	≥30	14	24.6%
Coitarche (Age at first sexual intercourse)	≤15	4	7.0%
	16–19	25	43.9%
	20–24	22	38.6%
	25–29	5	8.8%
	≥30	1	1.8%
Menarche (Age at first menstruation)	≤10	2	3.5%
	11–15	34	59.6%
	≥16	21	36.8%
Occupation	Artisan	5	8.7%
	Civil servant	6	10.5%
	Clergy	3	5.3%
	Unemployed	3	5.3%
	Trading	40	70.1%
Marital status	Divorced	6	10.5%
	Married	34	59.6%
	Widow	17	29.8%
Education level	Nil	14	24.6%
	Primary	12	21.0%
	Secondary	22	38.6%
	Tertiary	9	15.8%

**Table 2.** Clinical distribution.

Category	Interval	Count	Percentage
FIGO cancer staging	Stage 1A	0	0.0%
	Stage IB	4	7.0%
	Stage IIA	21	36.8%
	Stage IIB	12	21.1%
	Stage IIIA	7	12.3%
	Stage IIIB	11	19.3%
	Stage IVA	2	3.5%
Anaemia	No	14	24.6%
	Yes	43	75.4%
Cancer stage	Early (stages I–IIA)	25	43.9%
	Late (stages IIB–IV)	32	56.1%
Postcoital bleeding	No	37	64.9%
	Yes	20	35.1%
Menopause	No	15	26.3%
	Yes	42	73.7%

**Table 3.** Measure of association.

Variable	Category	Late stage	Early stage	OR (95% CI)	*p*-value
Age group	≥50	24	18	1.17 (0.35–3.85)	1.00
	<50	8	7		–
Parity group	≤4	15	8	0.53(0.16–1.76)	0.30
	≥5	17	17		
Postcoital bleeding	Yes	11	12	0.57 (0.20–1.66)	0.34
	No	21	13		–
PCV distribution	≤19	9	8	0.83 (0.27–2.60)	0.55
	20–29	15	10	1.32 (0.46–3.80)	0.60
	≥30	11	4	2.75 (0.76–10.02)	0.16
Coitarche	≤15	2	2	0.77 (0.10–5.88)	1.00
	16–19	17	8	2.41 (0.81–7.17)	0.11
	20–24	14	8	1.65 (0.55–4.91)	0.37
	25–29	2	3	0.49 (0.08–3.17)	0.68
	≥30	0	1	–	–
Menarche	≤10	3	2	1.19 (0.18–7.73)	1.00
	11–15	19	12	1.58 (0.55–4.53)	0.40
	≥16	14	7	2.00 (0.65–6.11)	0.22
Education level	Nil	12	3	**4.40 (1.08–17.82)**	**0.045**
	Primary	7	6	0.89 (0.26–3.07)	0.82
	Secondary	13	8	1.45 (0.48–4.35)	0.45
	Tertiary	3	4	0.54 (0.11–2.69)	0.70
Anaemia	Yes	24	19	0.95 (0.35–3.85)	1.00
	No	8	6	Reference	–
Menopause	Yes	29	13	**4.46 (1.27–15.70)**	**0.014**
	No	5	10	Reference	–

Bold values indicate statistically significant associations at *p* < 0.05.

**Table 4.** Measure of association (Crude and AOR).

Variable	Category	Late stage (*n*)	Early stage (*n*)	OR (95% CI)	AOR (95% CI)
Age group	≥50	24	18	1.17 (0.35–3.85)	1.10 (0.30–4.00)
	<50	8	7	Reference	Reference
Parity group	≤4	15	8	0.53 (0.16–1.76)	0.60 (0.18–2.00)
	≥5	17	17	Reference	Reference
Postcoital bleeding	Yes	11	12	0.57 (0.20–1.66)	0.60 (0.20–1.80)
	No	21	13	Reference	Reference
PCV distribution	≤19	9	8	0.83 (0.27–2.60)	0.95 (0.30–3.00)
	20–29	15	10	1.32 (0.46–3.80)	1.25 (0.40–3.90)
	≥30	11	4	2.75 (0.76–10.02)	2.50 (0.70–9.50)
Coitarche	≤15	2	2	0.77 (0.10–5.88)	0.80 (0.10–6.50)
	16–19	17	8	2.41 (0.81–7.17)	1.90 (0.70–5.15)
	20–24	14	8	1.65 (0.55–4.91)	1.50 (0.50–4.50)
	25–29	2	3	0.49 (0.08–3.17)	0.45 (0.07–3.00)
	≥30	0	1	–	–
Menarche	≤10	3	2	1.19 (0.18–7.73)	1.10 (0.15–8.00)
	11–15	19	12	1.58 (0.55–4.53)	1.50 (0.50–4.50)
	≥16	14	7	2.00 (0.65–6.11)	1.80 (0.60–5.40)
Education level	Nil	12	3	**4.40 (1.08–17.82)**	**3.20 (1.02–10.05)**
	Primary	7	6	0.89 (0.26–3.07)	0.85 (0.25–2.90)
	Secondary	13	8	1.45 (0.48–4.35)	1.30 (0.45–3.80)
	Tertiary	3	4	0.54 (0.11–2.69)	0.50 (0.10–2.50)
Anaemia	Yes	24	19	0.95 (0.35–3.85)	0.90 (0.30–2.70)
	No	8	6	Reference	Reference
Menopause	Yes	29	13	**4.46 (1.27–15.70)**	**3.80 (1.25–11.55)**
	No	5	10	Reference	Reference

Bold odds ratios (ORs) and adjusted odds ratios (AORs) indicate 95% confidence intervals that do not cross 1, corresponding to *p* < 0.05.
